# Comparative evolutionary analysis of protein complexes in *E. coli *and yeast

**DOI:** 10.1186/1471-2164-11-79

**Published:** 2010-02-01

**Authors:** Adam J Reid, Juan AG Ranea, Christine A Orengo

**Affiliations:** 1 Research Department of Structural & Molecular Biology, University College London, London, WC1E 6BT, UK; 2 Department of Molecular Biology and Biochemistry, University of Malaga, Malaga, Spain; 3 Current address: Wellcome Trust Sanger Institute, Wellcome Trust Genome Campus, Hinxton, Cambridge CB10 1SA, UK

## Abstract

**Background:**

Proteins do not act in isolation; they frequently act together in protein complexes to carry out concerted cellular functions. The evolution of complexes is poorly understood, especially in organisms other than yeast, where little experimental data has been available.

**Results:**

We generated accurate, high coverage datasets of protein complexes for *E. coli *and yeast in order to study differences in the evolution of complexes between these two species. We show that substantial differences exist in how complexes have evolved between these organisms. A previously proposed model of complex evolution identified complexes with cores of interacting homologues. We support findings of the relative importance of this mode of evolution in yeast, but find that it is much less common in *E. coli*. Additionally it is shown that those homologues which do cluster in complexes are involved in eukaryote-specific functions. Furthermore we identify correlated pairs of non-homologous domains which occur in multiple protein complexes. These were identified in both yeast and *E. coli *and we present evidence that these too may represent complex cores in yeast but not those of *E. coli*.

**Conclusions:**

Our results suggest that there are differences in the way protein complexes have evolved in *E. coli *and yeast. Whereas some yeast complexes have evolved by recruiting paralogues, this is not apparent in *E. coli*. Furthermore, such complexes are involved in eukaryotic-specific functions. This implies that the increase in gene family sizes seen in eukaryotes in part reflects multiple family members being used within complexes. However, in general, in both *E. coli *and yeast, homologous domains are used in different complexes.

## Background

Most proteins in cells carry out their function as subunits of protein complexes [1]. These aggregations range in size from 2 to >70 chains sometimes complexed with other types of molecules such as RNA and DNA. Small complexes often comprise multiple copies of the same protein but large complexes such as the ribosome tend to contain many different proteins. Complexes can be stable as in the case of the proteasome or transient as in the case of a kinase interacting with its substrate. The role of these high order structures is to coordinate complex processes which require the colocation of separate functional elements.

Dezso et al. [2] have shown that yeast protein complexes contain an essential, invariant core with irreplaceable biochemical function. The phenotype resulting from deletion of core proteins reflects the role of the complex as a whole. Furthermore recent work has suggested that complexes consist of cores, modules and attachments [3,4]. Gavin et al. [3] repeatedly purified hundreds of yeast complexes using Tandem Affinity Purification (TAP) and clustered the components based on their frequency of occurrence. Complex members were then classified into three groups: cores, attachments and modules. Core proteins were those which almost always appeared in a particular complex, attachments those which were less frequently observed. Modules were defined as groups of attachment proteins which always occurred together, often in different complexes. Functionally this suggests that attachment proteins are modifiers which are expressed at certain times to change aspects of complex function. A classic example of this is the variety of sigma factors available to bacterial RNA polymerase which alter its specificity for different promoter sequences [5].

It is currently unclear how well protein complexes are conserved between species. For a particular complex in one species, many species are deficient in some of the subunits [6]. Additionally there is a very low overlap in Protein-Protein Interactions (PPIs) detected between species [7] suggesting that PPIs may change rapidly during evolution [8], however this may also be due to a lack of experimental evidence. Recent work using combined PPI datasets suggests that pairs of complex members are well conserved between yeast and human [9]. Van Dam & Snel argue that PPIs between species rarely change within protein complexes but that complexes evolve through gain and loss of subunits. There is evidence that the Last Universal Common Ancestor (LUCA) contained protein complexes related to those of extant organisms [10].

The evolutionary conservation of some complexes has been examined in detail. Comparisons of the eukaryotic SWI/SNF and RSC chromatin remodelling complexes have shown that they consist of an evolutionarily conserved core of subunits [11]. Across eukaryotes there are variations in accessory subunits involved in these complexes. Some subunits, present in multiple species, may be necessary for organismal viability in one case but not another. Two contrasting modes of complex evolution are shown by the eukaryotic and prokaryotic NADH:Ubiquinone oxidoreductase, also termed complex I. While the early prokaryotic complex is thought to have formed from the combination of small pre-existing complexes [12], it appears that the eukaryotic complex tripled in size by step-wise recruitment of individual new subunits [13].

Many small complexes observed in structural data are homodimers and this arrangement confers several advantages. Firstly, homodimers can evolve stable interactions more parsimoniously than heterodimers [14]. Secondly, producing larger complexes from a single component rather than multiple components allows for greater genetic efficiency, requiring only a single gene and regulatory mechanism. It has been proposed that some homomeric complexes have diverged by duplication of the gene encoding the self-interacting protein [15]. The duplication of such a gene allows for divergence of one partner resulting in functional diversification and asymmetrical gain and/or loss of interactions in the complex. It has been shown that paralogues in the same complex perform different roles [16]. The F1 ATP synthase and the RecA recombinase homohexamer are examples of complexes which appear to have evolved in this manner, probably from the same homomeric ancestor [17]. There is evidence for between one tenth and a third of complexes in yeast having evolved in this way depending on the dataset considered [15].

There has been much discussion about whether duplicates deriving from Whole Genome Duplication (WGD) adopt different fates than those deriving from individual duplication events. Wapinski et al [18] showed that WGD rarely leads to paralogous modules. However, duplication of complexes has been shown to be important in yeast [19]. These are thought to have similar general function but novel specificities. They rarely duplicate in their entirety, but more commonly in a partial, stepwise fashion [16,18].

Datasets of protein complexes fall into four categories. Those arguably most accurate are the relatively small curated datasets provided for yeast by the MIPS [20] resource and for *E. coli *by EcoCyc [21]. Complexes derived from structural data (e.g. Protein Quaternary Structure database [22]) are also thought to be very accurate, although again relatively low in coverage and also biased towards stable interactions. Tandem Affinity Purification linked to Mass Spectrometry (TAP-MS) is a high-throughput experimental approach for identifying protein complexes. Large-scale datasets have been produced for yeast [3,23] and *E. coli *[24,25]. Such datasets cover a greater proportion of interactomes than curated or structural data. These TAP datasets have been used to generate datasets of protein complexes using computational methods [3,23]. The fourth source of complex data comprises a range of approaches for computationally inferring complexes from protein-protein interaction data. Resources such as IntAct [26], MINT [27] and BIND [28] provide datasets of protein-protein interactions in a range of species, derived from various low and high-throughput experiments including TAP-MS. It has been shown that protein complexes can be accurately inferred from experimental data [29]. Genetic interaction data [30] and predicted interactions such as those found in the STRING database [31] have also been used [32].

The *in silico *study of protein complexes has largely focussed on yeast where there are more data than for other organisms. Many of these studies have used structural and/or TAP-MS complexes [e.g. [15,33]]. Several authors [29,33,34] have also explored complexes derived from Protein-Protein Interaction Networks (PINs) using clustering methods. This results in larger datasets of complexes, with greater coverage of genomes than are available from other sources. This is achievable because PINs have highly connected regions which have been shown to correlate with complexes [35]. Several different clustering methods have been applied to the task of identifying complexes in PINs. Markov CLustering algorithm [MCL; [36]] uses flow simulation in graphs to detect clusters and was used by Pereira-Leal et al. [34] and Krogan et al. [23]. Pereira-Leal et al. showed that the clusters were functionally coherent in terms of regulatory and metabolic annotation, cellular localisation data and known complexes. MCODE [35] uses local neighbourhood density to define clusters. Both Netcarto [37] and Restricted Neighbourhood Search Clustering (RNSC) [38] use a cost function and Monte Carlo methods to obtain a division of the graph. Netcarto was used by Tamames et al. [33] to explore the relationship between reduction in genome size and network modularity. Super-Paramagnetic Clustering (SPC) [39] has also been applied to this problem. An analysis of several of these methods by Brohee & van Helden [29] showed that MCL was the best overall method for determining known yeast complexes from PPI datasets.

The evolution of protein complexes is still poorly understood and differences between species have been difficult to study on a global scale. In order to probe the differences in complex evolution between species we created protein complex datasets for a prokaryote and a eukaryote: the gram-negative bacterium *Escherichia coli *and the single-celled eukaryote *Saccharomyces cerevisiae*. For these species we derived combined PPI datasets using experimentally determined interactions from the Intact [26] and MINT [27] resources. MCL was used to derive protein complexes which we show to be accurate representations of known complexes, although coverage was noticeably lower for *E. coli *than for yeast.

We used the datasets clustered by MCL to examine the distribution of homologues amongst protein complexes showing that they tend to be randomly distributed in both species. Non-randomly distributed homologues tended to be involved in eukaryote-specific complexes such as the spliceosome and proteasome. We show that complex evolution in *E. coli *differs from yeast with a smaller proportion of complexes containing cores of homologous protein pairs. Furthermore we show that both *E. coli *and yeast have complexes which share duplicate, non-homologous protein pairs. There is evidence that these pairs may also form cores of yeast complexes, but not those of *E. coli*.

## Results & Discussion

### Determination and functional characterisation of protein complexes in *E. coli *and yeast

#### Prediction of protein complexes by clustering Protein Interaction Networks

In order to study the evolution of protein complexes we wanted to use accurate datasets with high genome coverage. We used an approach similar to that employed by Brohee & van Helden [29], Pereira-Leal et al. [34] and Lubovac et al. [40]. Protein-Protein Interactions (PPIs) for a particular species e.g. yeast, were combined into a Protein Interaction Network (PIN) and clustered using the MCL algorithm (see methods). The MCL algorithm has been shown to be the best amongst several approaches available for clustering PINs into complexes [29]. The MCL clustering algorithm requires a parameter to control the granularity of clusters known as the inflation parameter, *I*. We optimised this parameter on the yeast PIN by determining accuracy against the MIPS dataset of known yeast complexes as was done by Brohee & van Helden [29], using the same measure of accuracy (see methods).

**Figure 1:**
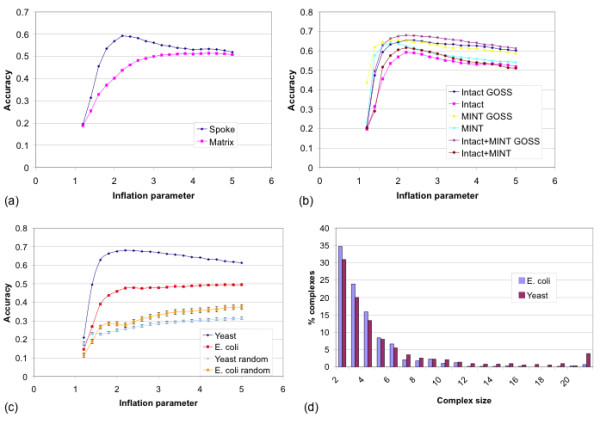
**Generation of MCL-GO complex datasets**. (a) For IntAct data, rendering TAP-MS data using the spoke model rather than the matrix model gave improved performance. (b) Combining IntAct and MINT datasets and weighting interactions with GOSS scores gives greater accuracy over either resource alone and without weighting. (c) Accuracy of MCL-GO complexes (using MINT+IntAct and edge weighting) in capturing MIPS yeast complexes and EcoCyc *E. coli *complexes. 'Random' lines show mean accuracy achieved over 10000 sets of randomised clusters. Error bars show one standard deviation either side of the mean. (d) Size distribution of *E. coli *and yeast MCL-GO complexes.

Two sources of PPI data were considered in generating complexes: MINT [27] and IntAct [26]. TAP-MS data, a component of both MINT and IntAct, identifies a complex between one 'bait' protein and several 'prey' and it is necessary to apply one of two models to generate pairwise interactions which are required for the clustering step. The spoke model specifies an interaction between the bait and each of the prey, whereas the matrix model additionally specifies interactions between each pair of prey proteins. Therefore the matrix model supposes that the proteins interacting with the bait protein all interact with each other. TAP-MS data from MINT had already been converted to pairwise interactions using the matrix model however TAP-MS data from IntAct was still in the one-to-many form. Figure 1a shows that, where a choice of models could be applied, the spoke model gave higher accuracy in identifying known yeast complexes from MIPS [20]. This suggests that the spoke model may be most appropriate for clustering TAP-MS data into complexes. The spoke model was subsequently applied to all TAP-MS data from IntAct. A dataset (MINT+IntAct) was then created by taking the union of pairwise interactions in the MINT and IntAct datasets. This resulted in six sets of pairwise interactions: MINT, IntAct and MINT+IntAct each for yeast and *E. coli*.

Where possible each interaction was then weighted using the semantic similarity of the biological process GO terms of the corresponding proteins (see methods). This is a measure of the functional similarity of two proteins in terms of their role in the cell. Figure 1b shows that, for yeast, the combined MINT+IntAct dataset with weighted edges resulted in the most accurate complexes compared to other datasets with unweighted edges. We refer to this approach as MCL-GO and datasets derived from it as MCL-GO datasets.

Figure 1c shows that the optimal clustering parameter for reproducing yeast MIPS complexes was I = 2.2, similar to the value of 1.8 found to be optimal by Brohee & van Helden [29] on a different dataset. The accuracy achieved, 0.68, is comparable to that achieved in recent studies [23,41].

Figure 1c also shows the accuracy of *E. coli *MCL-GO complexes in reproducing the known *E. coli *complexes from EcoCyc. The optimal value of I was also 2.2. Although there is a slight increase in performance at higher inflation parameter values the separation from random is much greater at I = 2.2. We have observed an accuracy for *E. coli *complexes which is noticeably lower than yeast, although still very much above random. This might be caused by lower coverage of the *E. coli *genome with PPIs. We therefore go on to test our key hypotheses using several pre-existing datasets.

The MCL-GO clusters for each species were filtered to remove clusters containing only one protein. This resulted in 574 *E. coli *complexes containing a total of 2210 distinct protein sequences and 855 yeast complexes containing a total of 4740 distinct protein sequences. These complex datasets thus covered roughly 56%, and 85% of *E. coli *and yeast genomes respectively based on genome sizes of 3952 and 5586 genes (genome sizes were taken from Integr8 [42]). Figure 1d shows the size distribution of complexes. Although the distributions are very similar between both species, yeast complexes were on average larger than *E. coli *complexes.

#### Functional classification of predicted protein complexes

**Figure 2:**
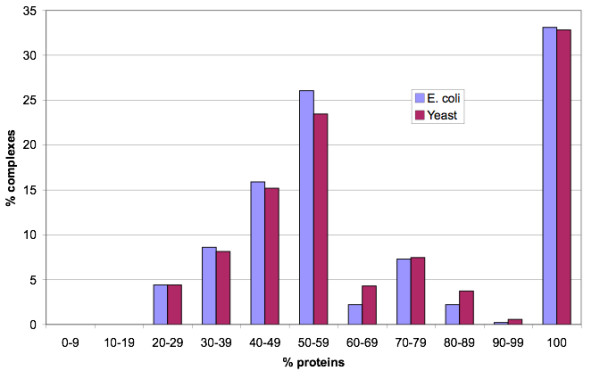
**Coverage of complexes by single functional terms**. This figure shows the percentage of proteins in yeast MCL-GO complexes which could be annotated with the most common term in each complex. Complexes were classified using FunCat terms. Complexes with <2 annotated proteins were excluded.

To determine whether the MCL-GO complex datasets made biological sense we analysed their functions. Although we have used functional terms (Gene Ontology) to generate the complexes, we felt it was important to determine whether they represented tight functional units. We have done this using FunCat terms rather than GO to try to reduce circularity, however we accept that there may be some bias. In any case we believe it is interesting to examine the functional distribution of complexes in our datasets. Figure 2 shows the percentage of proteins which are annotated with the most common FunCat term in their complex. For both *E. coli *and yeast around half of complexes were completely covered by only one term. The majority of proteins (>50%) could be described by a single functional term in ~75% of *E. coli *and yeast complexes. These results suggest that the MCL-GO complexes tend to be functionally coherent, with the majority of proteins in the majority of complexes performing the same general function. Furthermore it suggests that in both species, complexes can be reasonably well annotated using the most frequent term applied to its constituent proteins.

**Figure 3:**
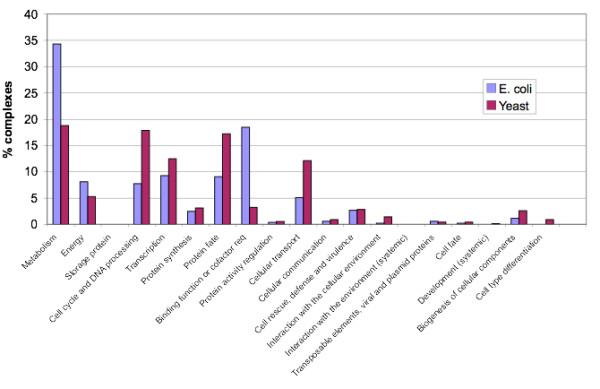
**Principal function of MCL-GO complexes in each species**. Complexes were classified using FunCat terms. Complexes with <2 annotated proteins were excluded.

We annotated each complex using its most common FunCat term. Figure 3 shows the proportion of complexes in each species that are involved in different processes. *E. coli *had a larger proportion of complexes devoted to metabolism and energy than yeast whereas yeast had a greater proportion of complexes involved in the cell cycle, transcription and cellular transport. Cell cycle and transcriptional processes are more complex in eukaryotes due to DNA packaging in chromosomes and intricate regulation respectively. Thus the MCL-GO complexes for *E. coli *and yeast appear to reflect the known biology of these species.

We also compared the distribution of functions for *E. coli *and yeast MCL-GO complexes to the curated EcoCyc and MIPS complexes. The figures are presented in additional file 1. They reveal that there may be certain functional biases in the smaller, curated datasets reflecting biases in the cellular processes best studied in *E. coli *and yeast.

### Distribution of protein domain superfamilies amongst protein complexes

There has been much debate about the fate of duplicated genes. It has been proposed that newly duplicated gene products initially retain common interactions which subsequently diverge [43]. There have been conflicting reports however regarding the extent to which paralogues within species tend to have common interactions and how fast they might lose these during evolution [43,44]. We wanted to determine how homologues are distributed in protein complexes and how this might relate to complex evolution. To define homologues we used CATH domain superfamilies which are based on structural data and capture distant evolutionary relationships.

**Figure 4:**
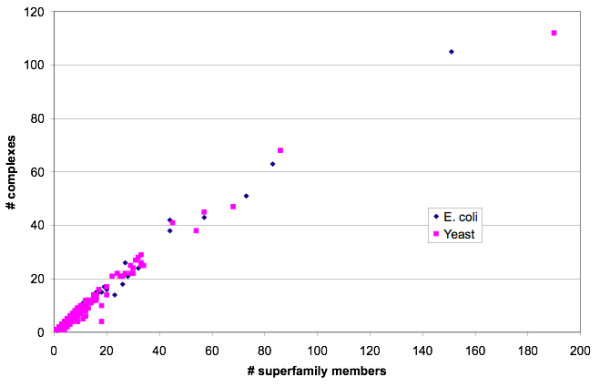
**Distribution of CATH superfamilies in MCL-GO complexes**. This figure shows the number of CATH superfamily members versus number of complexes containing members of that superfamily for *E. coli *and yeast MCL-GO complexes.

Figure 4 shows, for *E. coli *and yeast MCL-GO complexes, the number of superfamily members against the number of different complexes in which each superfamily is found. There was a strong positive correlation between superfamily size and the number of complexes in which that superfamily was found. For *E. coli *r^2 ^is 0.99 and for yeast 0.97. This suggests that duplicate domains do not tend to conserve their functional context.

Are there superfamilies which do not follow this trend and tend to conserve their complex membership? For each superfamily we determined how many times two proteins, both containing a member of that superfamily, were found together in a complex. This was compared to the number of co-complex pairs that would be expected if the proteins were distributed randomly amongst complexes (see methods). We found that, for most superfamilies, their members did not co-occur in complexes more than would be expected by chance. 98% of *E. coli *superfamilies and 95% of yeast superfamilies were randomly distributed. The exceptional, non-randomly distributed superfamilies are discussed in the next section.

We wanted to know whether different members of a superfamily were involved in similar biological processes despite their random distribution amongst complexes. Using *biological process *Gene Ontology terms we found that 28% of superfamilies in *E. coli *and 22% in yeast had members which were involved in more similar biological processes than expected by chance (p < 0.01). While homologous domains tend to become involved in different complexes after duplication, 1/5 to 1/3 of superfamilies appeared to conserve their functional role to some extent. However, when we compared the functional similarity of the proteins with which each superfamily member was directly interacting, there was much less conservation (1% of E. coli and 8% of yeast superfamilies having interactors with conserved function), i.e. if protein A interacts with proteins B, C and D and protein A homologue A' interacts with E, F and G, then B, C and D are not functionally similar to E, F and G. This suggests that those superfamilies which conserve their function to some extent tend to diversify into distinct aspects of similar processes. This has been recognized previously in the work of Baudot et al. [44]. Here we find that the trend is stronger in *E. coli *than in yeast, although the functional data we have used is less reliable in *E. coli*. Further details of this analysis are presented in additional file 1.

### Functional analysis of non-randomly distributed superfamilies

A small number of superfamilies were found to be non-randomly distributed amongst MCL-GO complexes in the previous analysis; Table 1 shows details of these superfamilies. What is the functional significance of multiple homologues within a complex?

**Table 1:** Non-randomly distributed superfamilies in *E. coli *and yeast MCL-GO complexes.

	Superfamily	Frequency	Function	Species distribution
***E. coli***				
	NAD(P)-binding Rossmann-like Domain (3.40.50.720)	73	Oxidoreductase activity in a wide variety of processes	Universal

**Yeast**				
	RNA binding (2.30.30.100)	11	RNA binding/splicing	Universal
	Glutamine Phosphoribosylpyrophosphate, subunit 1, domain 1 (3.60.20.10)	18	Ubiquitin-mediated endopeptidase activity	Universal
	Quinoprotein amine dehydrogenase (2.130.10.10)	68	Wide range of activities including protein synthesis	Universal
	Protein tyrosine phosphatase superfamily (3.90.190.10)	12	Dephosphorylation in signalling pathways	Eukaryotic
	Ribosomal Protein (3.30.1370.10)	4	Binding activity in a variety of processes	Universal
	Ubiquitin-like superfamily (3.10.20.30)	5	TCA cycle	Universal

CATH codes are shown in brackets. Frequency values are the number of proteins containing a member of that superfamily in that complex dataset. Functional descriptions are based on the most common GO terms from proteins containing the superfamily in that particular organism. Superfamilies are considered to belong to a kingdom when they are found in at least 70% of completed genomes from that kingdom. Universal refers to eukaryotes, eubacteria and archaea.

In *E. coli *there was only one non-randomly distributed superfamily identified, the NAD(P)-binding Rossmann-like Domain superfamily. This is a very large, universal (present in all three superkingdoms) domain superfamily which provides oxidoreductase activity in a wide variety of biological processes. Those complexes containing multiple members of this superfamily tended to be large, with diverse functional roles. It was therefore unclear as to the role of multiple members of this superfamily in individual complexes.

In yeast there were six non-randomly distributed superfamilies amongst MCL-GO complexes. These fell into three categories. The first is RNA processing. The *RNA-binding *superfamily was found in two complexes relating to the spliceosome. The spliceosome is a complex which removes introns from pre-mRNA and requires functions which include binding a variety of RNAs. Multiple members of the *Quinoprotein Amine Dehydrogenase Domain *superfamily were found in complexes rich in annotation relating to the spliceosome in one case and rRNA processing in the other. The *Ribosomal Protein *superfamily was found in a complex rich in annotation for rRNA processing. rRNA processing is known to occur in the nucleolar complex which is involved in the production of ribosomes. However associations related to rRNA processing may represent a bias in some of the experimental data used to generate the complexes. Some high-throughput complex identifications in yeast [45,46] contain many complexes erroneously enriched in rRNA processing due to various regions connected by rRNA, rather than protein interactions [47]. Independent evidence supports the relevance of the spliceosome however [48].

The second category is the proteasome. The *Glutamine Phosphoribosylpyrophosphate *superfamily is involved in Ubiquitin-mediated endopeptidase activity via the proteasome complex and different members of the superfamily are required for different types of protease activity [49].

The third category is signal transduction. Multiple copies of the *Protein Tyrosine Phosphatase *superfamily are found in a complex involved in signal transduction via a MAP kinase pathway controlling pseudohyphal growth. There was no clear role for the *Ubiquitin-Like *superfamily.

It appears that those members of superfamilies which cluster together tend to be involved in eukaryote-specific processes. They are however almost exclusively universal superfamilies (common to prokaryotes, eukaryotes and archaea), suggesting that these eukaryotic advancements have largely developed from duplication and divergence of pre-existing superfamilies. Here we have described the functions of complexes to better understand the importance of co-complex paralogues. In the spliceosome and proteasome variations on similar functions such as RNA binding and proteolysis are required. Multiple copies of homologous regulatory proteins may represent alternative regulatory subunits of signalling/regulatory complexes e.g. the Myc-Max and Mad-Max basic-helix-loop-helix transcription factor complexes noted by Pereira-Leal et al. [15]. In MCL-GO complexes, complex variants with alternative regulatory subunits will tend to be found as single complexes. In a case where protein A interacts with proteins B and C but never at the same time the clustering procedure used may result in a complex containing A, B and C. Mutual exclusivity of different interactions is not captured in current interaction data and each protein can only occur in a single complex in the procedure used in our work.

### Co-occurrence of homologous domains in protein complexes

We have shown that homologous domains tend to be randomly distributed amongst protein complexes and that duplicates therefore tend to diversify rather than remain involved in the same complex. An alternative analysis by Pereira-Leal et al. [15] has shown that interacting, homologous pairs might be important for complex evolution in yeast. They found that 10-30% of complexes in this species contain homologous protein pairs. In the model of complex evolution they presented, the gene encoding a homodimer duplicates and diverges resulting in a paralogous, heterodimeric protein complex. Rather than examine the distribution of individual domain or protein families in distinct complexes, they considered what proportion of complexes contained homologous pairs. We have used this complex-wise approach to further explore differences between *E. coli *and yeast.

**Figure 5:**
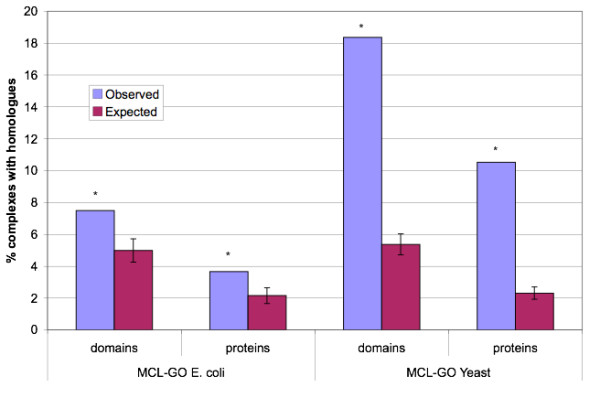
**Percentage of MCL-GO complexes containing homologous pairs**. Homologous pairs are defined here as either homologous domains shared between two proteins or proteins sharing a common domain architecture. All observed values were significantly larger than expected (p < 0.01).

For each predicted *E. coli *or yeast complex we determined whether there was at least one pair of proteins sharing, in the first case, a homologous domain or, in the second case, their entire multi-domain architecture (Figure 5). If there is a tendency for homologous proteins to occur together in complexes more than expected by chance, then these may be involved in the model of complex evolution described by Pereira-Leal et al. [15] and above. Using individual domains allows distant relationships to be identified which might otherwise be obscured by gain or loss of domains. By comparing the MCL-GO complexes with randomised complexes we take into account differences in frequency of homologous domain pairs between *E. coli *and yeast. We found that the proportion of complexes containing homologues was greater than expected by chance in each species (p < 0.01). In *E. coli *7.5% of complexes contained homologues at the domain level; 1.5 times more than expected by chance. There were 516 pairs of homologues co-occurring in *E. coli *complexes and these pairs were found to interact significantly more often than random pairs of proteins from the same complex (p = 0.001). For yeast the value was much higher and we observed 18.4% of complexes which contained homologues, 3.4 times more than expected. 720 pairs of co-complex homologues were identified in yeast and these were significantly enriched for interactions (p = 0.001). Furthermore these pairs were found to be co-expressed to a greater degree than expected by chance giving further support to their concerted role in complexes (p < 0.001; see additional file 1 for details). The result for yeast was within the bounds of the result of 10-30% suggested by Pereira-Leal et al. *E. coli *had a much smaller proportion of complexes which can have evolved from interacting paralogues. 43 complexes were identified in *E. coli *compared to 157 in yeast.

The trends between species in terms of relative numbers of complexes involved and the difference between expected and observed counts are similar when considering proteins sharing at least one homologous domain (domain homologues) or those sharing entire multi-domain architectures (protein homologues).

To determine whether the trend for fewer complexes containing homologues in *E. coli *was influenced by the way in which the complexes were generated, the frequency of homologues in the PINs of the two species were also examined. This showed the same trend, with a smaller percentage of links between homologues in *E. coli *than yeast. This suggests that a lower frequency of interacting homologues in *E. coli *is a feature of interactions in general, not just those within complexes. Details are presented in additional file 1.

**Figure 6:**
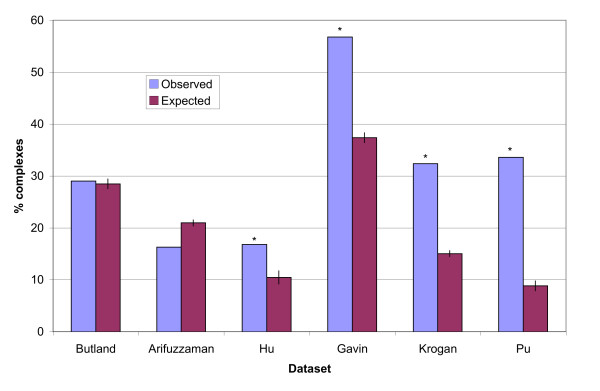
**Percentage of complexes containing homologous pairs for alternative datasets**. Asterisks show observed values which were significantly above random.

We also examined several alternative complex datasets to determine whether they supported our conclusions (Figure 6). Four of these datasets (Butland, Arifuzzaman, Gavin and Krogan) were derived from only TAP-MS data. These datasets, although lower coverage than our MCL-GO complex datasets, allow individual proteins to occur in multiple complexes, which our clustered datasets do not. Butland and Arifuzzaman *E. coli *complexes showed no significant increase in the number of complexes containing homologous pairs relative to random complexes. However the Gavin and Krogan Exp-TAP yeast complexes showed significant proportions of complexes containing homologous pairs. Near the end of our study two further datasets providing useful comparison were published. Hu et al. [50] published a new dataset of *E. coli *protein complexes created by clustering high-confidence PPIs. Pu et al. [51] published a new, curated complex dataset for yeast more comprehensive than that from MIPS. Analysis of these additional datasets confirms the trend we identified in MCL-GO complexes for yeast. In *E. coli*, the TAP-MS datasets provide no evidence for homologous pairs having a significant role in *E. coli *complex evolution, however the dataset of Hu suggests that some homologous pairs may have a small role as was found using the *E. coli *MCL-GO dataset.

### Identification of correlated domain superfamily pairs

We have shown that homologous domain pairs are a less common feature of protein complexes in *E. coli *than in yeast, and reaffirmed (after Pereira-Leal et al. [15]) that homologous domain pairs are not present in the majority of complexes. We wanted to determine the relative importance of non-homologous domain pairs which co-occur in multiple complexes. Such pairs have been identified previously by Betel et al. [47] in yeast. We wanted to determine whether they might represent an alternative route of complex evolution to that of paralogous heteromers.

189 pairs of correlated superfamilies were identified in *E. coli *MCL-GO complexes, involving 156 superfamilies. These pairs occurred in 68 separate complexes (~12%). This is a greater proportion of complexes than that containing paralogous pairs (~8%). In yeast MCL-GO complexes, 183 pairs were identified, involving 186 superfamilies and 83 complexes (~10%). Full details of the superfamily pairs identified are presented in additional file 1. We determined whether these superfamily pairs tended to interact more often than expected by chance using IntAct and MINT PPI datasets. In *E. coli *and yeast there was a significant tendency for interaction (p < 0.001). In both species the pairs were also significantly more functionally similar (using biological process Gene Ontology terms as described in Methods) than expected by chance (p < 0.001). Furthermore the pairs tended to have more highly correlated expression than expected by chance (p < 0.001; see additional file 1 for details). These results suggest that, in both *E. coli *and yeast, protein pairs with correlated domains have a tendency to be more functionally similar than random pairs of proteins in the same complex.

17% (114) of superfamilies in *E. coli *and 20% (127) in yeast were involved in correlated superfamily pairs. 39 of these superfamilies were involved in correlated pairs in both species, however none of the superfamily pairs were found to be correlated in both species. This suggests that these pairs may not persist over long evolutionary timescales.

### Do homologous and correlated domain pairs correspond to complex cores?

Having determined that both species have protein complexes with correlated domain pairs, we wanted to determine the role of these pairs in the complexes. In particular we were interested to see whether these pairs might represent cores of complexes. In order to do this we used the same method as Pereira-Leal et al. [15]who showed that homologous pairs represent complex cores of some yeast complexes. This analysis determines whether an arbitrary set of proteins tend to be older than other proteins. Specifically we determined the species distribution of the orthologues of proteins containing correlated domains to ascertain whether these proteins tended to emerge earlier in evolution. Older proteins are more likely to represent evolutionary conserved complex cores, whereas more recently evolved proteins are likely to represent later modifications to complexes [9,15]. We also looked at the age of interacting, homologous domain pairs to determine whether those that do occur in *E. coli *represent complex cores or whether there is further evidence against this mode of evolution in this species.

Although there is a tendency for orthologues of interacting homologous pairs from *E. coli *to be present in more distantly related organisms than other proteins (Table 2), this trend was not found to be significant (p = 0.09). This is further evidence that *E. coli *complexes have not evolved from interacting homologues, at least not to the extent seen in yeast. The same was true of proteins containing correlated domains (p = 0.28), suggesting that they are not associated with evolutionary cores of complexes.

**Table 2:** Relative age (emergence of orthologues) of all proteins, interacting homologues and proteins which contain correlated domains for *E. coli *and yeast MCL-GO complexes

	All proteins	Interacting homologous pairs	Non-homologous correlated pairs
***E. coli***			
*E. coli *K12-specific	19.0%	9.0%	11.6%
Proteobacteria	21.0%	10.4%	12.5%
Proteobacteria Firmicutes	7.8%	9.0%	5.4%
Bacteria	1.4%	3.0%	1.8%
Eukaryota+Bacteria	25.1%	29.9%	37.5%
Bacteria+Archaea	7.3%	10.4%	8.0%
Universal	18.4%	28.4%	23.2%
**Yeast**			
*S. cerevisiae*-specific	44.8%	13.1%	12.1%
Fungi	11.1%	9.3%	12.1%
Fungi + Metazoa	7.4%	10.4%	7.9%
Eukaryotes	10.3%	23.5%	14.3%
Eukaryotes + Archaea	4.2%	9.7%	10.0%
Eukaryotes + Bacteria	13.2%	18.3%	26.4%
Universal	9.0%	15.7%	17.1%

Interacting, homologous proteins in yeast were found to be significantly older than proteins in general (p < 0.01). This supports the result of Pereira-Leal et al. [15] using more recent datasets. It can also be seen that, in yeast, proteins containing correlated domains are significantly older than proteins in general (p < 0.01). Most of these proteins were found in all types of eukaryotes, whereas yeast proteins in general tended to be no older than the split between metazoa and fungi. This suggests that both interacting proteins with homologous domains and entirely non-homologous proteins with correlated domain pairs are involved in evolutionary cores of yeast protein complexes.

Table 3 shows that the *E. coli *Exp-TAP datasets support the trends identified in the MCL-GO dataset. Neither proteins with homologous domains nor correlated domain pairs were significantly older than other proteins in the Arifuzzaman [25] and Butland [24] Exp-TAP *E. coli *datasets. The picture is less clear in the yeast Exp-TAP datasets. Although the Krogan dataset supports the finding that correlated domains are older than other proteins, the test for homologous pairs was not quite significant. In the Gavin Exp-TAP [3] yeast dataset neither type was significant. These analyses are detailed in S5. Although positive trends in yeast were not found in all cases these results provide further evidence for a distinction between the two species.

**Table 3:** P-values indicating groups of proteins significantly older than other proteins.

Exp-TAP Dataset	Homologous Pairs (p-value)	Correlated Pairs (p-value)
E. coli (MCL-GO)	0.09482	0.2807
E. coli (Arifuzzaman)	0.881	0.913
E. coli (Butland)	0.818	0.670
Yeast (MCL-GO)	9.55E-05	6.95E-05
Yeast (Gavin)	0.388	0.282
Yeast (Krogan)	0.053	0.006

## Conclusions

We have presented an analysis of the distribution of homologous domains in the protein complexes of *E. coli *and yeast. In order to achieve this, protein complex datasets were generated and shown to accurately reproduce known complexes.

We found that homologous protein domains tend to be randomly distributed amongst complexes and therefore tend to occupy distinct functional niches. Those exceptional superfamilies whose members were found together more than expected by chance were involved in signalling/regulation or a limited number of eukaryote-specific complexes requiring colocation of similar functions. It has been shown that homologues are rarely found together in small molecule metabolic pathways of *E. coli*[52] and we have shown that this is also the case for protein complexes, in both *E. coli *and yeast.

Pereira-Leal et al. [15] proposed that a proportion of yeast complexes have evolved from cores of homologous subunits. These subunits are proposed to originate from a homodimer, encoded by a single gene which then duplicated, resulting in a dimer of paralogues. Our results suggest that this model of complex evolution might be limited to eukaryotes. We found that in *E. coli *there were far fewer complexes which could have evolved in this way. It is known that there is less gene copy redundancy in prokaryotes and that their gene families are smaller [53] resulting from streamlined genomes [54]. We show that this may extend to fundamental differences in how complexes have evolved in these organisms. These results were consistent with our functional analysis which showed that those homologues which cluster in complexes tend to relate to eukaryotic functions. This process may therefore have been exploited principally in developing the more complex processing and regulation required in the eukaryotic cell.

We then identified pairs of correlated domains which occur together in multiple complexes as was done previously by Betel et al. [47]. It was shown that the proteins containing these domains tended to interact and be more functionally similar than other pairs of co-complex proteins. In yeast these protein pairs tended to be older than other pairs of proteins and might therefore represent complex cores; there was little evidence for this in *E. coli *however. Complexes are known to have duplicated in yeast and these correlated pairs are likely to include parts of duplicated complexes. There has been a whole genome duplication in yeast [55], but not in *E. coli *[56], however it is not thought that this resulted in duplicate complexes [18,40], but more likely that complexes have been duplicated in a stepwise fashion [16,19]. If *E. coli *complexes have changed relatively little over a large evolutionary timescale, cores would not be detectably older than other parts and they could not be distinguished by their age. This would account for a failure to identify correlated domains as *E. coli *complex cores.

There is currently less protein-protein interaction information available for *E. coli *than yeast and thus we can be less certain about our conclusions in this organism. We have however tested our key conclusions using several alternative datasets for both species and found similar results in every case.

In future studies it would be interesting to examine higher eukaryotes to determine whether these processes of complex evolution are more common than in yeast. *Drosophila melanogaster *was considered for our analysis; however there was insufficient data to produce a reliable complex dataset.

## Methods

### Experimental protein-protein interaction datasets used for generating MCL-GO complexes

Protein-Protein Interaction (PPI) datasets were generated by taking the union of interactions from the MINT [27] and IntAct [26] resources, as they existed in Gene3D v5 [57]. Much of the data from these resources is from high-throughput experiments such as Two-Hybrid and Tandem Affinity Purification (TAP) but is also derived from small-scale pull-down and co-immunoprecipitation experiments. Interactions derived from TAP-MS data are between one 'bait' protein and multiple 'prey' proteins. Pairwise PPIs can be extracted from this data using one of two models. The spoke model defines interactions between the bait protein and each of the prey. The matrix model however defines pairwise interactions between the bait and prey proteins and between each pair of prey proteins. TAP-MS data from MINT was already in the matrix form, however IntAct data could be converted into either. The spoke model was used as it was shown to perform best in replicating known complexes (Figure 1a). For *E. coli *(NCBI taxon id: 562) there were 13941 interactions between 2865 proteins (~72% genome coverage) and for *S. cerevisiae *(NCBI taxon id: 4932) 38825 interactions covering 5735 proteins (~100% genome coverage).

### Generating MCL-GO complex datasets from PPI datasets

The *E. coli *and yeast combined PPI datasets described above were clustered into complex datasets using the MCL algorithm [36]. It has been shown that enriching Protein Interaction Networks (PINs) with functional annotation improves detection of functional modules [40]. Complex datasets were generated with and without weighting of the PINs. Unweighted edges were set to one, weighted edges were set to one plus the Gene Ontology Semantic Similarity (GOSS) score. To generate these GOSS scores, proteins were annotated with GO biological process terms from Gene3D. The GO terms used were those described in 'Annotation of MCL-GO Complexes'. The terms were compared using the Resnik [58] method described by Lord et al. [59] to determine their functional similarity. Each edge in the network was weighted using the highest GOSS score between any pair of terms assigned to the relevant nodes. Complex datasets generated in this way are referred to as MCL-GO datasets.

The inflation parameter, which controls the granularity of the clusters produced, was optimised by comparing predicted complexes (clusters) with curated, *gold standard *complexes from MIPS in the case of yeast and EcoCyc in the case of *E. coli*. The comparison was performed in the same way as described by Brohee & van Helden [29], using the same measures of sensitivity, Positive Predictive Value (PPV) and accuracy. When calculating sensitivity and PPV, only those clusters which had at least one member of a known complex were considered, those without a complex label could not be used to determine accuracy.

Sensitivity is the weighted average over all complexes of the proportion of each gold standard complex *i *captured by the predicted cluster *j*, best reflecting that complex.

In the above formula *N*_*i *_is the number of proteins in complex *i *and  is the complex-wise sensitivity defined below.

The complex-wise sensitivity is the maximum sensitivity *Sn*_*i*, *j *_for a particular complex, taking the greatest value over all predicted clusters.

In the above formula *T*_*i*, *j *_is the number of members of complex *i *in cluster *j*. Sensitivity is calculated only considering complexes with more than 1 protein.

Positive Predictive Value (PPV) is a measure of how pure the predicted clusters are, i.e. for the most common complex type in each cluster, what percentage of labelled proteins in the cluster are from this complex?

Here *T*_*j *_is the number of members of cluster *j *with membership of a known complex and  is the cluster-wise PPV described below.

The cluster-wise PPV takes the maximum value of *PPV*_*i*, *j *_for a particular cluster over all complexes. *PPV*_*i*, *j *_is described below.

*T*_*i*, *j *_is the number of members of cluster *j *in complex *i*. The trade-off between sensitivity and PPV was captured by taking the geometric mean of the sensitivity and PPV, referred to as the accuracy (*Acc*).

The accuracy achieved on these datasets was compared to that for randomly generated complexes to show that our procedure was useful, as was done by Brohee & van Helden [29]. This was achieved by clustering PPI datasets with MCL, then shuffling proteins between complexes while preserving complex size and benchmarking the resulting complexes. For each value of the MCL inflation parameter, randomisations were performed 10000 times.

### Annotation of MCL-GO complexes

CATH [60] protein domain superfamily annotation was extracted from Gene3D v5 to allow homologous relationships between proteins to be identified. 2190 CATH domains were identified from 656 superfamilies in the 2210 proteins from the *E. coli *MCL-GO complexes, covering 1579 proteins (71%). The yeast MCL-GO complexes had 2666 CATH domains from 630 superfamilies over 2070 proteins (44% of protein in this dataset). Throughout this work, multiple members of the same superfamily within protein chains were ignored.

Functional data in the form of Gene Ontology [61] annotation was also extracted from Gene3D v5. For *E. coli*, coverage with GO terms derived from experimental annotation was very low so Electronically Inferred Annotation (IEA) was included, only negative results (ND - No biological Data available) were excluded. This resulted in 3989 biological process terms over 1803 proteins (82% coverage). For yeast MCL-GO datasets, IEA terms were ignored. This resulted in 10622 terms over 3926 proteins for yeast (83% coverage).

FunCat [62] functional terms were extracted from Gene3D v5. Only the most general (level 1) terms were considered. These were used to annotate MCL-GO complexes as FunCat provides a suitable set of high level terms. There were 12257 terms covering 1573 *E. coli *proteins (71% coverage) and 12385 terms covering 3432 proteins in yeast (72% coverage).

### Pre-existing protein complex datasets

#### Curated datasets used to validate predicted complexes

Pre-existing complex datasets were used in our analyses. High-quality, curated datasets of known complexes were required in order to determine how accurately PPI datasets could be clustered into complexes. Such datasets were available from EcoCyc [21] for *E. coli *and from MIPS [20] for yeast. The EcoCyc complexes comprised 232 non-redundant, multi-subunit complexes containing a total of 586 distinct protein sequences. The MIPS complexes comprised 192 non-redundant, multi-subunit complexes containing a total of 1036 distinct protein sequences.

#### Experimental datasets used to assess trends

MCL-GO complexes, derived by clustering PPIs from a variety of experimental approaches, were used throughout this work as they had higher coverage of the genomes of each organism than curated datasets or individual experimental approaches such as TAP. The clustering approach used however only allowed each protein to exist in a single complex. In reality some proteins exist in multiple complexes and this discrepancy could bias our results. Therefore we also examined complexes based only on TAP data, which does allow individual proteins to appear in multiple complexes. TAP experiments identify relationships between one 'bait' protein and multiple 'prey', directly inferring complexes without the need for clustering. These are referred to collectively as Exp-TAP datasets. *E. coli *Exp-TAP complex datasets were derived from Butland et al. [24] and Arifuzzaman et al. [25] and downloaded from http://sunserver.cdfd.org.in:8080/protease/PPI/. Yeast Exp-TAP complexes derived from Gavin et al. [3] and Krogan et al. [23]were downloaded from BioGRID [63]. These Exp-TAP datasets are referred to as Butland, Arifuzzaman, Gavin and Krogan, respectively.

Experimental datasets were annotated with GO terms and CATH domains using the same protocols as for the MCL-GO complexes.

### Distribution of protein domain homologues in complexes

In order to examine the distribution of homologues in complexes, the distribution of each CATH domain superfamily was compared to that in randomised complexes. Only domain superfamilies with at least 5 members in different proteins were considered for this analysis to give the test sufficient statistical power. Of 656 superfamilies in *E. coli*, 101 had at least 5 members (62% of domains); of 630 in yeast 113 had at least 5 members (68% of domains). For each superfamily we determined the number of distinct pairs of proteins containing that superfamily which were found in the same complex. This was compared to the number of distinct pairs which were found together in 10000 randomised complex datasets. Complexes were randomised by shuffling members between complexes, retaining the complex size distribution. For each superfamily, p-values were calculated by determining the proportion of these 10000 randomised trials where the observed number of pairs was exceeded. The False Discovery Rate (FDR) correction for multiple hypothesis testing was applied (as described in [64]), using α = 0.01.

### Identification of complexes containing homologous pairs

Having asked whether superfamilies have members which tend to co-occur in complexes, we asked whether complexes tend to contain co-occurring superfamily members. In examining the proportion of complexes which contain homologues we considered both domain and protein homologues. Two proteins which shared a common CATH superfamily member were considered domain homologues. Two proteins which shared their entire CATH Multi-Domain Architecture (MDA) were considered protein homologues. A MDA is the series of domain annotations from N terminus to C terminus, excluding multiple domain segments, potentially domain-containing gaps and tandem repeats. We wanted to determine whether complexes tended to contain pairs of homologues and so the number of complexes which contained at least one pair of homologous proteins (using either domain or protein homologues) was counted. To determine whether the number of observed complexes was significant, the observed count was compared against the distribution of counts derived from 10000 randomised complex datasets. P-values were calculated empirically. Complex datasets were randomised by shuffling complex membership while retaining the size of complexes. We assume that these, largely interacting, homologues have resulted from homodimer duplication, which has been demonstrated by [65] to be a reasonable assumption.

### Identification of correlated domains

We identified pairs of CATH domain superfamilies (i.e. pairs of non-homologous domains) *A *and *B *such that *A *occurs in protein *p *and *B *occurs in protein *q *and *p *and *q *are present in the same complex. We then determined which of these pairs occurred in more complexes than expected by chance. This procedure was performed previously by Betel et al. [47]. For each domain pair occurring in at least two complexes, the frequency of occurrence was compared against frequencies found in 10000 randomised complex datasets and an empirical p-value calculated by determining in what proportion of these datasets the frequency of co-occurrence of the pairs exceeded that observed in the initial complex dataset. Those pairs with a p-value > 0.01 were excluded.

We wanted to determine whether proteins containing these correlated domain pairs tended to interact directly. We compared the frequency with which these proteins were observed to interact in MINT and IntAct data with the frequencies of interaction of the same number of randomly chosen co-complex protein pairs. Sets of random co-complex pairs were created 10000 times to derive a p-value. We also wanted to know whether correlated pairs represented functional units within complexes. To do this the average GOSS score between these pairs was compared with the average GOSS score between the same number of random co-complex protein pairs. Again this was performed 10000 times to derive a p-value.

### Phylogenetic profiling

We wanted to determine whether correlated domain pairs might represent protein complex cores. To do this we assume that proteins in the core of complexes are older than other proteins, i.e. their orthologues are found in more distantly related species. We used the analysis employed by Pereira-Leal et al. [15] to determine the age of protein orthologues. For any one protein it was determined in what group of organisms their orthologous group arose. Bi-directional best hit BLAST orthologues were determined for each *E. coli *and yeast protein amongst 32 species (listed in additional file 1). Orthologues were defined as bi-directional best hits between two species with an E-value of ≤0.01. The point of origin of a particular protein was defined by the age group in which an orthologue was found. Age groups were defined using the species tree of Baldauf [66].

The age groups defined for *E. coli *in this analysis were '*E. coli *specific', 'Proteobacteria', 'Proteobacteria/Firmicutes', 'Bacteria', 'Eukaryota+Bacteria', 'Bacteria+Archaea' and 'Universal'. For yeast we used '*Saccharomyces cerevisiae *specific', 'Fungi', 'Metazoa/Fungi', 'Eukaryota', 'Eukaryota+Archaea', 'Eukaryota+Bacteria' and 'Universal'. The chi-square test was used to determine whether significant differences existed in the age distribution of different classes of proteins.

## Authors' contributions

AJR designed the study and carried out the analyses. JAGR assisted in study design and helped draft the manuscript. CAO supervised the project and helped draft the manuscript. All authors read and approved the final manuscript.

## Supplementary Material

Additional file 1 **supplementary methods and results**. We analyse complex functions in MCL-GO and gold standard datasets showing potential biases in the gold standard datasets. We examine the functional coherence of superfamilies finding that yeast has more superfamilies which are involved in a wider range of biological processes, but are on average less diverse in terms of their catalytic actions or cellular locations. Furthermore we find that different members of the same superfamily carry out their functions in different contexts. We describe methods and detailed results for calculating correlated expression. We examine the frequencies of observed and expected interactions between homologues in protein interaction networks. The details of the phylogenetic profiling work is described. Click here for file
