# Supplementary material for: Comparative evolutionary analysis of protein complexes in E. coli and yeast
Source: BMC Genomics. 2010 Feb 1;11:79. doi: 10.1186/1471-2164-11-79 (PMC2837643; doi:10.1186/1471-2164-11-79)
Supplement: Additional file 1 — supplementary methods and results. We analyse complex functions in MCL-GO and gold standard datasets showing potential biases in the gold standard datasets. We examine the functional coherence of superfamilies finding that yeast has more superfamilies which are involved in a wider range of biological processes, but are on average less diverse in terms of their catalytic actions or cellular locations. Furthermore we find that different members of the same superfamily carry out their functions in different contexts. We describe methods and detailed results for calculating correlated expression. We examine the frequencies of observed and expected interactions between homologues in protein interaction networks. The details of the phylogenetic profiling work is described. [file 1471-2164-11-79-S1.DOC]

Additional File 1

Comparative evolutionary analysis of protein complexes in *E. coli* & yeast

**Adam J. Reid, Juan A. G. Ranea and Christine A. Orengo**

Table of contents

S1. Comparison of complex functions in MCL-GO and gold standard datasets [2](#__RefHeading___Toc125514748)

S2. Functional coherence of superfamilies [4](#__RefHeading___Toc125514749)

S3. Correlated expression of homologous and correlated protein pairs in yeast [7](#__RefHeading___Toc125514750)

S4. Incidence of homologous domain pairs in PINs [8](#__RefHeading___Toc125514751)

S5. Phylogenetic profiling of interacting homologue and correlated protein pairs [9](#__RefHeading___Toc125514752)

S6. Species used in phylogenetic profiling analysis [13](#__RefHeading___Toc125514753)

## S1. Comparison of complex functions in MCL-GO and gold standard datasets

We analysed the functional distribution of MCL-GO complexes, as described in the main paper. Here, in addition we compare the functional distribution of these complexes with that of complexes from the gold standard datasets. Figure S1a shows that the *E. coli* MCL-GO complexes contain a higher proportion of complexes involved in metabolism, cell cycle and DNA processing, transcription, protein synthesis, protein fate and binding suggesting that such complexes are under-represented amongst known complexes. Figure S1b suggests that MIPS complexes are under-represented in metabolism, binding, cellular transport, cell rescue, interaction with the cellular environment and biogenesis of cellcular components. Interestingly the under-represented categories largely do not overlap between *E. coli* and yeast, perhaps representing a differential bias in the processes which are commonly studied in these organisms.

(a)

(b)

Figure S1. Functional distribution of complexes in (a) E. coli MCL-GO and Ecocyc complexes, (b) yeast MCL-GO and MIPS complexes.

## S2. Functional coherence of superfamilies

In the paper we determined that the majority of CATH superfamilies are randomly distributed in protein complexes. We wanted to determine whether, despite this, the members of a superfamily tended to retain similar functional roles.

For each superfamily we determined the functional coherence of proteins containing a member of that superfamily and compared it to random groups of proteins of the same size as the superfamily. Functional coherence was calculated as the average GOSS score between each pair of proteins, either containing the superfamily of interest or in the random group. Superfamilies were considered if they had at least 5 members and as least two members had relevent GO annotation. GOSS scores were calculated using biological process GO terms as specified in the main text. The percentage of superfamilies that were significantly more functionally coherent than expected are shown in Table S1. The table shows a higher proportion of superfamilies are conserved in their biological processes in *E. coli* than yeast. Conversely fewer superfamilies are conserved in molecular function and cellular component in *E. coli* than yeast. Notice that the numbers are correlated with organismal complexity. The results suggest that more complex organisms have superfamilies which are involved in a wider range of biological processes, but are on average less diverse in terms of their catalytic actions or cellular locations. In reality the superfamilies in more complex organisms may be just as mechanistically diverse, but they are larger and there is probably more redundant function, which is then used in a more diverse range of processes. However the numbers may relate to a role for superfamily expansions in eukaryotes to increase the number of biological processes, while expansion in prokaryotes may be more focussed on increasing metabolic complexity.

|  | Biological Process | Molecular function | Cellular component |
| --- | --- | --- | --- |
| *E. coli* | 28% (60/217) | 42% (94/225) | 0% (0/122) |
| Yeast | 22% (67/302) | 55% (163/294) | 12% (37/311) |

Table S1. Percentage of superfamilies which are more functionally coherent than expected by chance for each species and each part of the GO classification.

We also examined the conservation of function amongst the interactors of proteins containing a particular superfamily, e.g. do the interactors of one superfamily member perform similar functions to those of another superfamily member? The results are shown in Table S2. There is generally poor conservation of the functional neighbourhood for CATH domain superfamilies. There are especially few superfamilies in *E. coli* whose members have significantly similar functional neighbourhoods in its interaction network. A greater proportion of yeast superfamilies have conserved functional neighbourhoods, 10 times as many as in *E. coli*. The general lack of functional neighbourhood conservation in comparison to functional conservation within superfamilies themselves suggests that even in those superfamilies which are functionally conserved, the interactors of different superfamily members tend to have different functions. Thus, different superfamily members carry out their functions in different contexts.

For each superfamily, GOSS scores were calculated between each of its direct interactors and the interactors another member of that superfamily. The interactors of each superfamily member were compared against the interactors of every other one. The average GOSS score was taken between each superfamily pair and then the average of all of these comparisons. Proteins containing the superfamily of interest were excluded. This average was compared against the distribution of means derived by comparing 10000 randomised complexes of the same number and size (excluding the number of occurances of the query superfamily). The False Discovery Rate (FDR) was controlled by choosing only superfamilies with p-value ≤ ((k * α) / m), a less conservative approach than the Bonferroni correction for multihypothesis testing; α was set to 0.01.

|  | Biological Process | Molecular Function | Cellular Component |
| --- | --- | --- | --- |
| *E. coli* | 1% (1/101) | 1% (1/101) | 1% (1/101) |
| Yeast | 8% (9/114) | 8% (9/114) | 4% (5/114) |

Table S2. Percentage of superfamilies whose members interactors have conserved function

## S3. Correlated expression of homologous and correlated protein pairs in yeast

It was determined whether pairs of homologous proteins and pairs of proteins containing correlated domains had higher correlated expression than expected by chance. Correlated expression data for 6178 ORFs in yeast from the Spellman dataset (Spellman et al., 1998) was used to compare expression values from either test dataset to the population using the approach of Grigoriev (Grigoriev, 2001). The population mean was 0.033, standard deviation 0.215 and standard error 4.912x10^-5^. The mean for homologous pairs was 0.259 with a standard deviation of 0.289 and a standard error of 0.01 with p-value ~0. For the correlated pairs, the mean was 0.078, standard deviation 0.216 and standard error 0.015 with p-value <0.0001.

## S4. Incidence of homologous domain pairs in PINs

Figure S2. The percentage of interactions in the combined MINT and Intact PINs for *E. coli* and yeast. The same trend is observed as for complexes, with a greater proportion of interactions in yeast being between homologues than in *E. coli*.

## S5. Phylogenetic profiling of interacting homologue and correlated protein pairs

Table S3 shows the age of proteins in each MCL-GO and TAP complex dataset. Interacting homologues were found to be significantly older than other proteins (p ≤ 0.01) in the MCL-GO yeast dataset but not the TAP datasets. Proteins containing correlated domains were found to be significantly older than other proteins in the MCL-GO and Krogan complexes datasets.

| Dataset | All proteins % | | All proteins count | Interacting homologues % | Interacting homologues count | Correlated % | Correlated count |
| --- | --- | --- | --- | --- | --- | --- | --- |
| **E. coli MCL-GO** |  | |  |  |  |  |  |
| Escherichia coli K12 specific | 18.95573 | | 501 | 8.955224 | 6 | 11.60714 | 13 |
| Proteobacteria | 20.96103 | | 554 | 10.44776 | 7 | 12.5 | 14 |
| Proteobacteria Firmicutes | 7.832009 | | 207 | 8.955224 | 6 | 5.357143 | 6 |
| Bacteria | 1.43776 | | 38 | 2.985075 | 2 | 1.785714 | 2 |
| Eukaryota+Bacteria | 25.08513 | | 663 | 29.85075 | 20 | 37.5 | 42 |
| Bacteria+Archaea | 7.302308 | | 193 | 10.44776 | 7 | 8.035714 | 9 |
| Universal | 18.42603 | | 487 | 28.35821 | 19 | 23.21429 | 26 |
| P-value against all proteins |  | |  | 0.09482 |  | 0.2807 |  |
| Arifuzzaman | |  |  |  |  |  |  |
| Escherichia coli K12 specific | | 50.7734 | 1313 | 50.81081 | 94 | 48.95688 | 352 |
| Proteobacteria | | 13.8051 | 357 | 8.108108 | 15 | 8.901252 | 64 |
| Proteobacteria Firmicutes | | 4.679041 | 121 | 3.783784 | 7 | 4.172462 | 30 |
| Bacteria | | 0.812065 | 21 | 1.621622 | 3 | 1.668985 | 12 |
| Eukaryota+Bacteria | | 14.9652 | 387 | 17.83784 | 33 | 17.94159 | 129 |
| Bacteria+Archaea | | 4.563032 | 118 | 4.864865 | 9 | 5.006954 | 36 |
| universal | | 10.40217 | 269 | 12.97297 | 24 | 13.35188 | 96 |
| P-value against all proteins | |  |  | 0.8807 |  | 0.9128 |  |

| **Dataset** | **All proteins %** | **All proteins count** | | **Interacting homologues %** | **Interacting homologues count** | | **Correlated %** | | **Correlated count** | |
| --- | --- | --- | --- | --- | --- | --- | --- | --- | --- | --- |
| **Butland** |  |  | |  |  | |  | |  | |
| Escherichia coli K12 specific | 49.90584 | 530 | | 41.1215 | 44 | | 42.42424 | | 140 | |
| Proteobacteria | 11.67608 | 124 | | 10.28037 | 11 | | 7.575758 | | 25 | |
| Proteobacteria Firmicutes | 3.389831 | 36 | | 2.803738 | 3 | | 2.727273 | | 9 | |
| Bacteria | 1.224105 | 13 | | 1.869159 | 2 | | 2.727273 | | 9 | |
| Eukaryota+Bacteria | 17.3258 | 184 | | 20.56075 | 22 | | 24.54545 | | 81 | |
| Bacteria+Archaea | 4.613936 | 49 | | 4.672897 | 5 | | 3.939394 | | 13 | |
| universal | 11.86441 | 126 | | 18.69159 | 20 | | 16.06061 | | 53 | |
| P-value against all proteins |  |  | | 0.8178 |  | | 0.6697 | |  | |
| **MCL-GO yeast** |  |  |  | | |  | |  | |  |
| Saccharomyces cerevisiae specific | 44.75737 | 2066 | 13.0597 | | | 35 | | 12.14286 | | 17 |
| Fungi | 11.11352 | 513 | 9.328358 | | | 25 | | 12.14286 | | 17 |
| Metazoa Fungi | 7.387348 | 341 | 10.44776 | | | 28 | | 7.857143 | | 11 |
| Eukaryota | 10.33362 | 477 | 23.50746 | | | 63 | | 14.28571 | | 20 |
| Eukaryota+Archaea | 4.246101 | 196 | 9.701493 | | | 26 | | 10 | | 14 |
| Eukaryota+Bacteria | 13.17158 | 608 | 18.28358 | | | 49 | | 26.42857 | | 37 |
| universal | 8.990468 | 415 | 15.67164 | | | 42 | | 17.14286 | | 24 |
| P-value against all proteins |  |  | 9.55E-05 | | |  | | 6.95E-05 | |  |

| **Dataset** | **All proteins %** | **All proteins count** | **Interacting homologues %** | **Interacting homologues count** | **Correlated %** | **Correlated count** |
| --- | --- | --- | --- | --- | --- | --- |
| **Gavin** |  |  |  |  |  |  |
| Saccharomyces cerevisiae specific | 22.31719 | 235 | 13.18681 | 36 | 12.32323 | 61 |
| Fungi | 9.97151 | 105 | 5.494505 | 15 | 5.252525 | 26 |
| Metazoa Fungi | 6.552707 | 69 | 4.395604 | 12 | 4.040404 | 20 |
| Eukaryota | 22.50712 | 237 | 32.23443 | 88 | 25.45455 | 126 |
| Eukaryota+Archaea | 11.39601 | 120 | 12.45421 | 34 | 15.35354 | 76 |
| Eukaryota+Bacteria | 14.81481 | 156 | 16.48352 | 45 | 18.9899 | 94 |
| universal | 12.44065 | 131 | 15.75092 | 43 | 18.58586 | 92 |
| P-value against all proteins |  |  | 0.3881 |  | 0.282 |  |
| **Krogan** |  |  |  |  |  |  |
| Saccharomyces cerevisiae specific | 30.86053 | 624 | 15.20468 | 52 | 13.63636 | 15 |
| Fungi | 12.46291 | 252 | 6.140351 | 21 | 7.272727 | 8 |
| Metazoa Fungi | 8.209693 | 166 | 7.017544 | 24 | 4.545455 | 5 |
| Eukaryota | 16.5183 | 334 | 26.02339 | 89 | 28.18182 | 31 |
| Eukaryota+Archaea | 6.03363 | 122 | 11.11111 | 38 | 16.36364 | 18 |
| Eukaryota+Bacteria | 14.93571 | 302 | 20.17544 | 69 | 13.63636 | 15 |
| universal | 10.97923 | 222 | 14.32749 | 49 | 16.36364 | 18 |
| P-value against all proteins |  |  | 0.05332 |  | 0.006202 |  |

Table S3. Age of proteins in MCL-GO and TAP complex datasets

## S6. Species used in phylogenetic profiling analysis

| Species | Classification | NCBI taxon Id |
| --- | --- | --- |
| *Oryza sativa* | Eukaryota; Viridiplantae; Streptophyta | 39947 |
| *Arabidopsis thaliana* | Eukaryota; Viridiplantae; Streptophyta | 3702 |
| *Dictyostelium discoideum* | Eukaryota; Mycetozoa; Dictyosteliida | 352472 |
| *Caenorhabditis elegans* | Eukaryota; Metazoa; Nematoda | 6239 |
| *Mus musculus* | Eukaryota; Metazoa; Chordata | 10090 |
| *Homo sapiens* | Eukaryota; Metazoa; Chordata | 9606 |
| *Danio rerio* | Eukaryota; Metazoa; Chordata | 7955 |
| *Anopheles gambiae* | Eukaryota; Metazoa; Arthropoda | 180454 |
| *Drosophila melanogaster* | Eukaryota; Metazoa; Arthropoda | 7227 |
| *Ustilago maydis* | Eukaryota; Fungi; Basidiomycota; Ustilaginomycetes | 5270 |
| *Saccharomyces cerevisiae* | Eukaryota; Fungi; Ascomycota; Saccharomycotina | 4932 |
| *Schizosaccharomyces pombe* | Eukaryota; Fungi; Ascomycota; Schizosaccharomycetes | 4896 |
| *Aspergillus fumigatus* | Eukaryota; Fungi; Ascomycota; Pezizomycotina | 5085 |
| *Plasmodium falciparum 3D7* | Eukaryota; Alveolata; Apicomplexa | 36329 |
| *Vibrio cholerae* | Bacteria; Proteobacteria; Gammaproteobacteria | 666 |
| *Pseudomonas putida KT2440* | Bacteria; Proteobacteria; Gammaproteobacteria | 160488 |
| *Haemophilus influenzae* | Bacteria; Proteobacteria; Gammaproteobacteria | 727 |
| *Yersinia pestis* | Bacteria; Proteobacteria; Gammaproteobacteria | 632 |
| *Escherichia coli K12* | Bacteria; Proteobacteria; Gammaproteobacteria | 562 |
| *Buchnera aphidicola (Bp)* | Bacteria; Proteobacteria; Gammaproteobacteria | 135842 |
| *Mycoplasma genitalium* | Bacteria; Firmicutes; Mollicutes | 2097 |
| *Clostridium acetobutylicum* | Bacteria; Firmicutes; Clostridia | 1488 |
| *Clostridium tetani* | Bacteria; Firmicutes; Clostridia | 1513 |
| *Bacillus subtilis* | Bacteria; Firmicutes; Bacillales | 1423 |
| *Thermus thermophilus HB27* | Bacteria; Deinococcus-Thermus; Deinococci | 262724 |
| *Synechococcus elongatus* | Bacteria; Cyanobacteria; Chroococcales | 32046 |
| *Mycobacterium tuberculosis* | Bacteria; Actinobacteria; Actinobacteridae | 1773 |
| *Nanoarchaeum equitans* | Archaea; Nanoarchaeota; Nanoarchaeum | 160232 |
| *Thermoplasma acidophilum* | Archaea; Euryarchaeota; Thermoplasmatasma | 2303 |
| *Pyrococcus furiosus* | Archaea; Euryarchaeota; Ther mococci | 2261 |
| *Methanocaldococcus jannaschii* | Archaea; Euryarchaeota; Methanococci | 2190 |
| *Aeropyrum pernix* | Archaea; Crenarchaeota; Thermoprotei | 56636 |

Reference List

Grigoriev,A. (2001) A relationship between gene expression and protein interactions on the proteome scale: analysis of the bacteriophage T7 and the yeast Saccharomyces cerevisiae. *Nucleic Acids Res.*, 29, 3513-3519.

Spellman,P.T. et al. (1998) Comprehensive identification of cell cycle-regulated genes of the yeast Saccharomyces cerevisiae by microarray hybridization. *Mol. Biol. Cell*, 9, 3273-3297.
